# Response to letter to the editor concerning “Short-term outcomes of anterior cruciate ligament reconstruction with or without lateral tenodesis or anterolateral ligament reconstruction: a retrospective cohort”

**DOI:** 10.1007/s00264-023-06001-7

**Published:** 2023-10-17

**Authors:** Ashraf T. Hantouly, Abdulaziz F. Ahmed, Theodorakys Marín Fermín, Luca Macchiarola, Vasileios Sideris, Emmanouil Papakostas, Pieter D’Hooghe, Khalid Al-Khelaifi, Bruno Olory, Bashir Zikria

**Affiliations:** 1 Department of Orthopedic Surgery, Hamad Medical Corporation, Doha, Qatar; 2 Department of Orthopaedic Surgery, Johns Hopkins School of Medicine, Baltimore, MD USA; 3 Aspetar Orthopaedic and Sports Medicine Hospital, Doha, Qatar; 4 Clinica Ortopedica E Traumatologica II, IRCCS Istituto Ortopedico Rizzoli, Via Pupilli 1, Bologna, BO Italy

Dear Editor,

We thank the authors for their observations and valuable discussion regarding our recent publication [1, 2]. In response, we have provided a detailed point-by-point response to their comments.

The comment regarding treatment algorithm and the influence of surgeons’ preference on graft type raises an important point. However, patients were allocated either based on appointment availability through a receptionist or as direct referrals to specific surgeons. While this allocation lacked randomization, it minimized the risk of selection bias significantly. Additionally, while we acknowledge this as a limitation, we emphasize the critical importance of aligning the surgical procedure with the surgeon’s knowledge, experience, and comfort. When surgeons perform techniques they are confident and familiar with, fewer complications and favourable outcomes can be attained. Therefore, guiding the treatment according to patients’ outcome was prioritized to minimize the bias of the study. Nevertheless, this point has been already highlighted as a study limitation to ensure transparency to the readers.

This study found a decrease of 1.1 in mean pain score at 6 weeks in patients who underwent a concomitant meniscal procedure compared to those who did not. However, longer follow-ups failed to demonstrate the same result. Ulstein et al. [3], in their prospective cohort study, proposed an improvement in pain scores from preoperative to five year follow-up in the concomitant meniscus procedures group. It is essential to acknowledge that our study had no preoperative scores, and the correlation, as stated in the manuscript, was not directly derived from our results, but was based on existing evidence [3].

About the ACL-RSI score, which consists of 12 items graded on a visual analogue scale from 0 to 100, we reported the scores without converting them to percentages, which does not have any effect on the results.

While the return to play is a valuable outcome, the primary focus of this study was the short-term outcomes. Additionally, there was significant variability in follow-up periods among the different techniques and a significant loss of follow-up at six and nine months. Consequently, reporting such outcome in such circumstances can lead to misleading and inaccurate reporting.

Regarding radiological outcomes, this study focused on the clinical aspect of the three techniques. While some studies utilized SNQ and other parameters to assess graft maturation, this study maintained a distinct clinical focus.

Indeed, the use of allografts may be required in some cases. However, their use is reserved for revisions and multi-ligament knee injuries. The ACL and ALL combined reconstruction described by Sonnery-Cottet et al. [4] does not require more graft removal than a simple hamstring ACL reconstruction. In this technique, the semi-tendinosous is used in three (or sometimes four) bands and the gracilis in a simple band, allowing an increase in the diameter of the intra-articular graft and, at the same time, rebuilding the ALL in the form of a tripod graft (only the posterior band is anatomical). As described by Sonnery-Cottet et al. [4], once again, the tibial insertion of the semi-tendinosous is preserved. At the same time, the gracilis is detached from its insertion to be sutured again on the semi-tendinosous a few centimeters from its distal insertion (this distance depends on the total length of the distance in between the tibial tunnel entry and the femoral tunnel exit). Thus, the gracilis allows not only to participate in the volume increase of the intra-articular graft but also in the reconstruction of the ALL. The performance of these gestures being percutaneous, the operative time is considerably reduced, explaining operating times comparable to those of an isolated ACLR. Of course, a learning curve will be necessary, both for the surgeon and for the entire surgical team.

We thank the authors for investing their time in reading and reviewing this publication. We also appreciate the peer review process as it undoubtedly improves research quality and ensures an accurate presentation of the results. However, it is essential to note that the authors’ comments do not impact the study’s results or conclusions.

## Data Availability

Not applicable.
